# The connection between nutrition education, behavior, and body composition in women following the Mediterranean diet: a sustainable approach

**DOI:** 10.1186/s12905-026-04454-7

**Published:** 2026-04-13

**Authors:** Ayşe Nur Kahve, Suhide Bilge Horzum, Melda Kangalgil

**Affiliations:** 1 Department of Sports and Health, Aksaray University, Aksaray, Turkey; 2 Department of Nutrition and Dietetics, Faculty of Health Sciences, Selçuk University, Konya, Turkey; 3 Department of Nutrition and Dietetics, Faculty of Health Sciences, Sivas Cumhuriyet University, Sivas, Turkey

**Keywords:** Anthropometric measurements, Nutrition education, Sustainable nutrition, Women’s health

## Abstract

**Background:**

This study aimed to examine the relationship between sustainable healthy eating behaviors and body composition in women who received sustainable nutrition education.

**Methods:**

The study was conducted with women aged 20–49 years attending a Nutrition and Diet Counseling Center. Participants were randomly assigned to either an intervention group, which participated in an 8-week sustainable nutrition education program delivered biweekly by a dietitian face-to-face, or a control group, which received traditional nutrition education throughout the study. Demographic data were collected at the beginning of the study. The Sustainable Eating Behavior Scale, which assesses sustainable eating behaviors, was collected at the beginning of the study and again at the end of the 8-week period, while anthropometric measurements were taken at the beginning and in the 4th and 8th weeks.

**Results:**

There is no statistical difference between the demographic and clinical characteristics of the two groups (*p* > 0.05). Significant decreases in weight, Body Mass Index, percent body fat, body fat mass, body muscle mass, and waist circumference were observed within the intervention and control groups over time (*p* < 0.001). The intervention group experienced greater mean decreases in weight, BMI, percent body fat, body fat mass, and waist circumference compared to the control group during the intervention period. Group-time interaction effects were found to be significant for body weight (F = 3.723, *p* = 0.027, η²=0.058) and BMI (F = 3.492, *p* = 0.034, η²=0.055).

**Conclusion:**

These results suggest that sustainable nutrition education can positively contribute to both sustainable eating behaviors and anthropometric parameters, highlighting its potential value in promoting healthier and more environmentally friendly dietary patterns.

**Trial registration:**

This study was registered at ClinicalTrials.gov (Registration number: NCT07247578, Registered on 15 November 2025). URL: https://clinicaltrials.gov/study/NCT07247578.

## Introduction

Increased consumption along with rapid population growth and the depletion of energy resources as a result of inefficient use of existing resources have necessitated the emergence of the concept of sustainability [1]. Food production, which is estimated to account for approximately 30% of total greenhouse gas emissions and consume approximately 70% of the world’s freshwater resources, places significant pressure on ecosystems and biodiversity [2]. Consequently, global problems such as hunger and malnutrition have become the most critical challenges of both today and the future [1].

Sustainable diets are diets that maintain ecological balance, are accessible, culturally acceptable, and affordable; are nutritionally adequate, safe, and healthy; and optimize the use of natural and human resources [3]. The concept of sustainable diets has become increasingly relevant due to the threats posed by global population growth and climate change. Food production and consumption are among the main driving forces of environmental degradation [4]. In recent years, studies investigating behaviors related to sustainable nutrition have shown a growing trend [5–8]. The United Nations Sustainable Development Goals (SDGs) aim to eradicate poverty, protect the planet, ensure prosperity for all, and eliminate hunger and malnutrition by 2030. However, the world is failing to make sufficient progress toward ensuring access to safe, nutritious, and adequate food for all and eradicating all forms of malnutrition [9]. According to the Sustainable Development Goals Report 2023, while some positive developments were made between 2015 and 2019, progress towards these goals has stagnated since 2020 due to the COVID-19 pandemic, economic crises, wars, and other factors. Turkey’s ranking of 72nd out of 166 countries on the SDG Index with an overall score of 70.8 demonstrates that it is at a significant point in improving sustainability [10]. Introducing consumers to sustainable dietary practices, which have positive impacts on both human health and the environment, is a crucial step in achieving this improvement. In this context, sustainable nutrition education is considered one of the first and most important tools for protecting the environment and health [11]. It has been stated that this education facilitates a better understanding of the concept of sustainable nutrition and helps individuals translate this knowledge into practical behavior [12]. Education can foster sustainable eating habits by encouraging individuals to seek and utilize new sources of information, including information about food and nutrition [6].

Among sustainable eating patterns, the Mediterranean diet is scientifically recognized as one of the oldest dietary patterns. It stands out as a health-promoting dietary model due to its distinctive characteristics, such as the consumption of unsaturated fats, the promotion of plant-based protein intake, low-glycemic carbohydrates, and high fiber and vitamin content [13]. Evidence suggests that adherence to the Mediterranean diet is associated not only with improved cardiovascular and metabolic outcomes but also with a reduced carbon footprint [14]. The Mediterranean Diet recommends consuming fish and seafood at least twice a week, white meat twice a week, eggs two to four times a week, red meat less than twice a week, one or fewer processed meats, and sweets less than three servings a week. On a daily basis, it suggests consuming two servings of dairy products, one to two servings of oily seeds and nuts, and higher amounts of spices, herbs, garlic, and onions. Each main meal should also include 1–2 servings of fruit, more than two servings of vegetables, olive oil, and 1–2 servings of whole grains [15]. Due to these health-beneficial components, the Mediterranean diet was inscribed on the Representative List of the Intangible Cultural Heritage of Humanity in 2013 [16].

The criteria for defining a sustainable and healthy diet remain unclear. However, some studies have identified several measurable elements of sustainable diets: (1) reducing overconsumption; (2) increasing the consumption of plant-based foods (vegetables, fruits, legumes and grains); (3) reducing the consumption of animal and processed foods; (4) focusing on seasonal and local produce; and (5) reducing food waste [17, 18]. The predominantly plant-based Mediterranean diet is known to have a relatively low ecological footprint [19]. Therefore, recent research has increasingly focused on the feasibility of the Mediterranean diet as a sustainable eating model [20].

In the literature, no studies have been found comparing individuals who receive nutrition education based on Mediterranean diet principles and sustainable nutrition education with those who receive traditional nutrition education, demonstrating the superiority of the former in terms of sustainable healthy eating behaviors and body composition. Therefore, the present study aims to compare the effects of sustainable nutrition education and traditional nutrition education on sustainable eating behaviors and anthropometric outcomes.

## Materials and methods

### Study population

This randomized controlled trial was conducted among women who presented to a Nutrition and Diet Counseling Center in the Selçuklu district of Konya after a physician screening. The study was conducted between July 2025 and September 2025. This study was registered retrospectively at ClinicalTrials.gov (Registration number: NCT07247578) on November 15, 2025, after the completion of data collection. Ethical approval for the study was obtained. Based on a significance level of 5% (α = 0.05), a large effect size (Cohen’s f = 0.4), and a statistical power of 80% (1-β = 0.80), it was calculated that a minimum of 60 participants at least 30 per group would be required for adequate statistical power [21, 22].

**Fig. 1:**
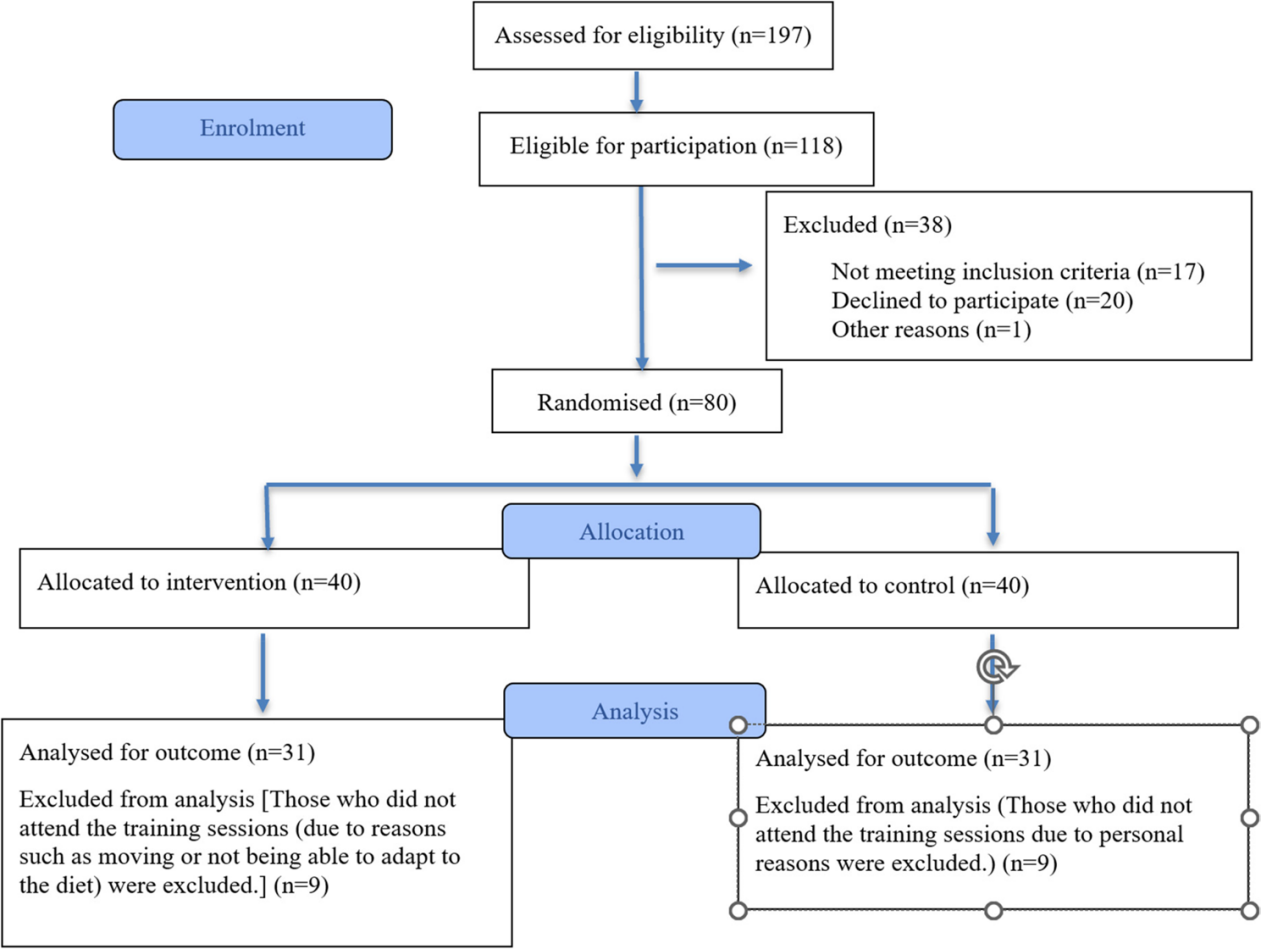
CONSORT 2025 flow diagram

The inclusion criteria for the study were as follows: being between 20 and 49 years of age; having a body mass index (BMI) between 25 kg/m² and 35 kg/m²; having no conditions that impair reality perception or cognitive functions, which could interfere with interviews or completion of questionnaires; being literate; and voluntarily agreeing to participate in the study by signing an informed consent form. Participants were excluded if they were pregnant or lactating; unable to attend one or more interview sessions; had an implanted pacemaker or defibrillator due to the potential theoretical interference caused by bioelectrical impedance measurements; had any chronic disease requiring a specific diet; regularly used medications that affect metabolism; were taking insulin or oral antidiabetic drugs; had an allergy or intolerance to any Mediterranean diet components (such as walnuts, hazelnuts, peanuts, almonds, etc.); were taking vitamin or mineral supplements; or had a physician-diagnosed psychiatric disorder. The participant flow diagram is presented in Fig. 1. This randomized controlled trial was reported in accordance with the CONSORT (Consolidated Standards of Reporting Trials) guidelines.

### Randomization and blinding

Participants were assigned a unique identification number generated via a computer-based system at the time of enrollment. These identification numbers were subsequently randomly allocated to either the intervention or control group using a computer-based randomization program. The researcher assigning participants was not aware of the group allocation at the time of enrollment, ensuring allocation concealment. Due to the nature of the intervention, participants could not be blinded; however, group allocation was not disclosed prior to assignment. Baseline similarity between groups further supports the effectiveness of the randomization process. After obtaining informed consent from each participant, an 8-week follow-up period was initiated.

### Intervention

**Fig. 2:**
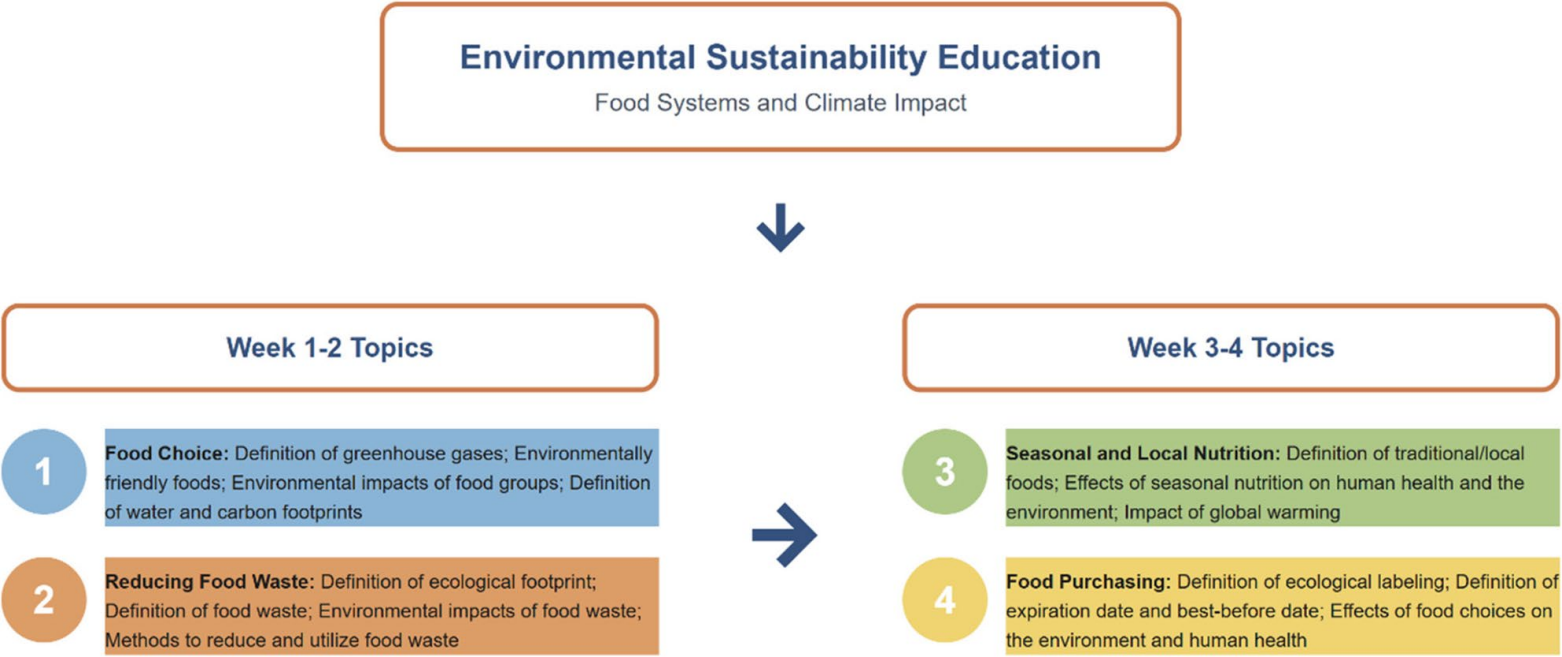
Sustainable nutrition education topics (The content of the education on nutrition [23])

After random assignment, women were divided into two groups: an intervention group that received sustainable nutrition education and a control group that received traditional nutrition education. Baseline data were collected, and the 8-week sustainable nutrition education program was initiated in the intervention group. The trained group received one hour of face-to-face sustainable nutrition education, delivered individually by a registered dietitian, once every two weeks. During the intervention phase, the dietitian supported the women in the intervention group using educational materials such as brochures and booklets. The training content was developed based on the principles of the Mediterranean diet; however, adherence to the Mediterranean diet was not assessed using a validated instrument. The intervention content included topics such as sustainable food choices, increasing the consumption of plant-based foods, reducing red and processed meat intake, minimizing food waste, choosing seasonal and local products, and adopting environmentally friendly dietary practices. Each session was structured to include both theoretical information and practical guidance aimed at facilitating behavioral change. A flow chart of the sustainable nutrition education topics is shown in Fig. 2.

The control group received traditional nutrition education delivered by the same dietitian, with the same frequency and duration as the intervention group (one-hour sessions every two weeks over 8 weeks). The content focused on general healthy eating recommendations without specific emphasis on sustainability principles. Following the 8-week training intervention, follow-up data were collected for the intervention and control groups. Following the completion of the training sessions, additional sustainable nutrition education sessions were held for the control group, in accordance with the principle of equality.

### Measures

#### Data collection tools

At the beginning of the study, participants were administered a 26-question questionnaire covering their general characteristics, dietary habits, health status, and anthropometric measurements, along with the Sustainable Eating Behavior Scale. The Sustainable Eating Behavior Scale was collected again at the end of the 8-week period, and anthropometric measurements were also taken in the 4th and 8th weeks of the study. 

##### Anthropometric measurements

Participants’ body weight and body composition were assessed using a foot-to-foot bioelectrical impedance analysis (BIA) device. Body fat percentage, body fat mass (kg), total body water (kg), waist circumference, visceral fat rating, metabolic age, waist-to-height ratio, and waist-to-hip ratio were measured using a TANITA MC-580 analyzer. Height Measurement; standing height was measured using a stadiometer with the participant in an upright position and the head aligned in the Frankfort horizontal plane (with the ear canal and the lower margin of the orbit in the same line and the gaze parallel to the floor). During the measurement, participants stood with their feet together and their back, hips, and heels touching the wall, and were asked to take a deep breath [23]. BMI was calculated as body weight divided by the square of height in meters and categorized according to the World Health Organization’s classification for adults [24].

##### Sustainable nutrition behavior scale

The Sustainable Nutrition Behavior Scale was developed by Garipoglu et al. [4], and its Turkish validity and reliability have been established. This instrument is a valuable tool for assessing sustainable nutrition behaviors among adults aged 18–65 years. The scale consists of 29 items and four subdimensions, formatted on a five-point Likert scale. Items 1–6 assess food preference, items 7–15 measure reduction of food waste, items 16–23 evaluate seasonal and local food consumption, and items 24–29 assess food purchasing behaviors. Response options range from “never” to “always,” and all items are scored positively on a scale from 1 to 5, beginning with “never.” The possible total score ranges from 29 to 145. Subdimension scores are calculated by dividing the sum of the item scores in each subdimension by the number of items in that subdimension. Higher total and subdimension scores indicate stronger sustainable nutrition behaviors.

#### Outcomes

The primary outcome of this study was the change over time in body composition parameters (body weight, BMI, body fat percentage, fat mass, muscle mass, and waist circumference) between the intervention and control groups. Secondary outcomes included changes in sustainable eating behaviors, as measured by the Sustainable Eating Behavior Scale.

### Statistical analysis

Statistical analyses were performed using Statistical Package for Social Sciences (SPSS, IBM, version 23). The normality of the data distribution was assessed using histogram plots, skewness and kurtosis values, and the Shapiro–Wilk test, while the Levene test was used to assess the homogeneity of variance. Continuous data with normal distributions were presented as means and standard deviations (SDs) and with nonnormal distributions as medians and interquartile ranges [IQR]. Categorical data are presented as the numbers (n) and percentages (%). The chi-square test and Fisher’s exact test were used to compare categorical variables. Continuous variables were analyzed using independent samples t-test or Mann-Whitney U test, as appropriate. Mixed-method repeated measures ANOVA was performed to test for differences in the parameters between the control and intervention groups and overtime. All statistical tests were two-tailed with a significant level of 0.05.

### Ethical considerations

The authors confirm that all procedures performed in this study adhered to the ethical principles established by national and institutional human research committees, in accordance with the Declaration of Helsinki (1975) and its 2013 revision. Ethical approval for all procedures involving human participants was obtained from the Scientific Research Ethics Committee of Aksaray University of Health Sciences, decision number 2025/172.

## Results

Baseline sociodemographic, nutritional and sleep characteristics are presented in Table 1. There were no statistical differences between the intervention and control groups for baseline socio-demographic, and nutritional characteristics (*p* > 0.05). The mean age of the participants in the intervention group was 40.3 years (SD = 11.5) compared to the control group at 40.0 years (SD = 11.8). Among the study participants, four reported smoking, whereas none reported alcohol consumption. All participants reported skipping meals at times, with the most common reasons being time constraints (45.2%) and weight loss (27.4%). There was no significant difference between the groups and behaviors scale towards sustainable nutrition (BSTSN) total scores (*p* = 0.549).

**Table 1:** Baseline characteristics of women

Characteristics	Control (*n* = 31)	Intervention (*n* = 31)	*p*
Age, year, M ± SD	40.0 ± 11.8	40.3 ± 11.5	0.931
Marital status, n (%)			
Single	7 (22.6%)	4 (12.9%)	
Married	24 (%77.4)	25 (80.6%)	0.147
Divorced	-	2 (6.5%)	
Job status, yes, n (%)	14 (45.2%)	8 (25.8%)	0.184
Education, n (%)			
Primary school	8 (25.8%)	15 (48.4%)	
Secondary school	5 (16.1%)	6 (19.4%)	0.180
High school	16 (51.6%)	8 (25.8%)	
Bachelor and higher	2 (6.5%)	2 (3.2%)	
Income status, n (%)			
Income is less than expenses	6 (19.4%)	5 (16.1%)	
Income equal to expenses	22 (71%)	23 (74.2%)	0.808
Income more than expenses	3 (9.7%)	3 (9.7%)	
Smoking, yes, n (%)	1 (3.2%)	3 (9.7%)	0.612
Main meal, median (Q1-Q3)	2 (2–3)	2 (2–3)	0.192
Snack, median (Q1-Q3)	1 (1–2)	2 (1–2)	0.076
Eating speed, n (%)			
Slow	1 (3.2%)	2 (6.5%)	
Normal	12 (38.7)	7 (22.6%)	0.515
Fast	18 (58.1%)	22 (71%)	
Previous diet history, yes, n (%)	11 (35.5%)	10 (32.3%)	0.788
Night eating behavior, yes, n (%)	2 (6.5%)	2 (6.5%)	1.000
Family history of obesity, n (%)	17 (54.8%)	22 (71%)	0.189
Sleep problems, yes, n (%)	14 (45.2%)	10 (32.3%)	0.297
Sleep duration, h, M ± SD	7.3 ± 1.3	7.2 ± 1.3	0.768
BSTSN total score, M ± SD	71.3 ± 20.6	74.8 ± 25.1	0.549

The primary outcome of this study was to evaluate the effect of an 8-week sustainable nutrition education program compared to traditional nutrition education intervention on body composition (Table 2). Both groups showed significant within-group changes over time in all measured parameters, including weight, BMI, body fat percentage, body fat mass, body muscle mass, and waist circumference (*p* < 0.001). Between-group comparisons were not statistically significant for any variable at any time point (*p* > 0.05). However, the intervention group experienced greater mean reductions in weight, BMI, body fat percentage, body fat mass, and waist circumference than the control group throughout the intervention period. The group-time interaction effects were significant for body weight (F = 3.723, *p* = 0.027, η² = 0.058) and BMI (F = 3.492, *p* = 0.034, η² = 0.055), indicating that changes in these parameters over time differed between the intervention and control groups. There were no significant group-time interaction effects for body fat percentage, body fat mass, muscle mass, or waist circumference (*p* > 0.05).

**Table 2:** Changes in body composition variables from baseline to follow-up

Variables	Group	T1 M ± SD	T2 M ± SD	T3 M ± SD	MD (T1-T2)	MD (T2-T3)	MD (T1-T3)	Between groups* F(p, η²)	Within group* F(p, η²)	Group-time effect* F(p, η²)
Weight, kg	Control	81.3 ± 16.1	78.5 ± 15.3	76.1 ± 14.5	2.852	2.416	5.268	2.287 (0.136; 0.037)	227.05 **(< 0.001**; 0.791)	3.723 (**0.027**; 0.058)
	Intervention	87.6 ± 13.6	83.9 ± 13.0	80.7 ± 12.8	3.658	3.158	6.816			
BMI, kg/m^2^	Control	32.1 ± 6.0	31.0 ± 5.8	30.1 ± 5.5	1.116	0.974	2.090	1.458 (0.232; 0.024)	241.88 **(< 0.001**; 0.801)	3.492 (**0.034**; 0.055)
	Intervention	34.2 ± 6.0	32.8 ± 5.7	31.5 ± 5.6	1.419	1.242	2.661			
Body fat percentage, (%)	Control	37.9 ± 5.2	36.6 ± 5.6	34.8 ± 6.0	1.277	1.819	3.097	1.225 (0.273; 0.020)	97.957 **(< 0.001**; 0.620)	0.057 (0.944; 0.001)
	Intervention	39.5 ± 6.1	38.4 ± 6.4	36.4 ± 6.7	1.145	1.948	3.094			
Body fat mass, kg	Control	31.5 ± 10.4	29.4 ± 9.9	27.2 ± 9.5	2.119	2.235	4.355	1.822 (0.182; 0.029)	223.19 **(< 0.001**; 0.788)	2.271 (0.108; 0.036)
	Intervention	35.4 ± 10.3	32.8 ± 10.1	30.1 ± 9.7	2.542	2.787	5.329			
Body muscle mass, kg	Control	47.3 ± 5.9	46.6 ± 5.9	46.1 ± 5.5	0.784	0.477	1.261	2.455 (0.122; 0.039)	17.753 (**< 0.001**; 0.228)	0.082 (0.922; 0.001)
	Intervention	49.5 ± 5.0	48.6 ± 4.7	48.2 ± 4.9	0.965	0.387	1.352			
Waist circumference, cm	Control	98.3 ± 15.8	95.2 ± 15.0	91.9 ± 14.3	3.097	3.323	6.419	2.298 (0.135; 0.037)	229.608 (**< 0.001**; 0.793)	2.276 (0.107; 0.037)
	Intervention	104.7 ± 15.0	100.9 ± 14.6	96.9 ± 14.0	3.839	4.000	7.839			

T1: Pre-intervention (baseline measurement). T2: Post-intervention (4 week). T3: Post-intervention (8 week). A p value <0.05 was considered statistically significant

Between groups indicates the main effect of the group factor (differences between intervention and control groups across time points)

Within group indicates the main effect of time (changes from pre-intervention to post-intervention average across groups)

Group-time effect represents the combined influence of group and time (differences in change patterns between groups)

*M* Mean, *SD* Standard deviation, *η²* partial eta-squared, *MD* Mean differences, *BMI* Body mass index

* The outcomes were based on mixed-method repeated measures ANOVA

**Fig. 3:**
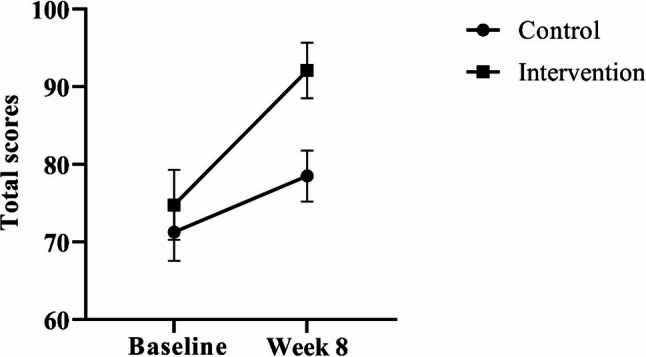
Behaviors Scale Towards Sustainable Nutrition (BSTSN) total scores (mean± standard error) at baseline and week 8 for the intervention and control groups

Figure 3 shows the total scores of participants on the(BSTSN in the control and intervention groups at baseline and after 8 weeks. At week 8, a statistically significant increase was observed in total score in the intervention group (MD = 17.290; 95% CI: 15.259–19.322) and the control group (MD = 7.258; 95% CI: 5.227–9.289; *p* < 0.001). Between-group comparisons of total scores from baseline to week 8 were not statistically significant (F = 2.546; *p* = 0.116; η² = 0.041). The group–time interaction effects were significant for total score (F = 48.793, *p* < 0.001, η² = 0.448) indicating that changes in these scores over time differed between the intervention and control groups.

## Discussion

Improving nutritional behaviors is essential for achieving the Sustainable Development Goals, improving overall public health, and planetary health. Sustainable nutrition education is seen as having potential impacts on preventing unhealthy eating habits [25]. It is known that general nutrition education has positive effects on increasing nutritional knowledge and changing both eating habits and anthropometric measurements. Studies evaluating the effects of sustainable nutrition education, beyond general nutrition education, are quite limited. Therefore, this study compared an 8-week sustainable nutrition education program received by women who received nutrition education based on Mediterranean diet principles with a traditional nutrition education program. At the beginning of the study, the intervention and control groups were similar in terms of demographic and behavioral characteristics, age, marital status, and eating behaviors (including meal frequency, snacking habits, eating pace, and nighttime eating habits) (Table 1). This strengthened the internal validity of the study. Furthermore, recent studies involving behavioral nutrition interventions have adopted similar characteristics between the intervention and control groups [26, 27].

Table 2 shows changes in body composition variables (weight, BMI, body fat percentage (%), body fat mass, body muscle mass, and waist circumference) from baseline to follow-up. Accordingly, when the effects of an 8-week intervention involving nutrition education based on Mediterranean diet principles and sustainable nutrition education on body composition were examined, significant improvements were observed in both the control and intervention groups. Significant decreases in body composition variables such as weight and BMI in both the intervention and control groups, and a significant group-by-time interaction, indicate that the intervention program produced significant effects. This result is supported by recent research, which has reported that sustainable nutrition education facilitates adherence to the Mediterranean diet by influencing individuals’ food choices, resulting in clinically significant weight loss even in the short term [28, 29].

In this study, it was determined that muscle mass in the intervention and control groups decreased slightly over time. The literature also indicates a slight loss of muscle mass due to energy restriction in dietary intervention studies [30]. Previous studies indicate that weight loss due to dieting will result in muscle loss unless supported by resistance exercise [31, 32]. Another study involving nutritional education reported no change in muscle mass [33]. Although a slight decrease in muscle mass was observed in the present study, this finding is consistent with previous literature [34]. The magnitude of this reduction was relatively small and may not be clinically significant. Therefore, the minimal muscle loss observed, particularly in the intervention group, can be considered a relatively positive outcome. However, future interventions should consider incorporating physical activity components, such as resistance exercise, to preserve lean mass.

The current study observed a decrease in waist circumference in both the control and intervention groups. The group-by-time interaction also yielded near-significant results, suggesting that sustainable nutrition education has a significant effect on abdominal fat. A study in which 294 participants were given a Mediterranean diet and followed for 18 months found a significant decrease in visceral fat [35]. In another study, university students received a 1-hour sustainable nutrition education program for 6 weeks and observed reductions in body weight, BMI, and fat mass [33]. In this study, the reduction in fat mass in the intervention group was greater than in the control group, but this was not statistically significant. However, the approximately 1 kg greater fat loss in the intervention group may be clinically significant. Time interactions were also found to be similar between groups. While sustainable nutrition education appears to have no immediate effect on fat loss, there are studies in the literature reporting that changes in body composition become evident with long-term follow-up [36, 37].

Few studies have examined the effectiveness of intervention programs that promote consumer dietary behavior change and sustainable nutrition, which has become an important area of research today [38]. Therefore, the current study presents the changes in BSTSN total scores from baseline to week 8, as shown in Fig. 3. Accordingly, a significantly greater increase was observed in the intervention group compared to the control group. No significant difference was found in baseline scores between the intervention and control groups, suggesting that sustainable eating behaviors were similar at baseline. This suggests that the positive changes in sustainable eating behaviors at the end of the 8-week period were related to the intervention itself, not to preexisting behavioral differences. In a study that continued sustainable nutrition education with 12 participants over a year, the intervention had a positive impact on overall dietary behavior. They also reported reducing food waste, opting for minimally packaged, seasonal foods, and prioritizing equitably sourced foods [39]. In a study conducted parallel to ours, participants were divided into a sustainable diet group (intervention) and a diet al.igned with current healthy eating guidelines (control). Participants decreased their red meat intake and increased their vegetable intake, including plant-based foods like beans, peas, and lentils, with significantly greater increases in the intervention group compared to the control group [40]. In a study conducted by Ghammachi et al. [41], they demonstrated that 3–12 week digital education programs promoting sustainable diets rapidly impacted nutritional behaviors. Therefore, the results of the 8-week sustainable nutrition education intervention in the current study, which showed significant improvements in behavioral scales, are consistent with the literature.

## Conclusion

This randomized controlled study is demonstrating that an 8-week sustainable nutrition education program intervention resulted in positive changes in sustainable dietary behaviors and various anthropometric measurements among women aged 20–49. Although differences in anthropometric measurements between the control and intervention groups were not statistically significant, the intervention group consistently showed greater reductions in body weight, BMI, body fat percentage, body fat mass, and waist circumference compared to the control group. Additionally, a strong group-by-time interaction effect was observed for both BSTSN total scores and anthropometric measurement results, indicating that the intervention significantly contributed to behavioral and physiological improvements over time. This information suggests that sustainable nutrition education provided by a dietitian can help individuals determine their food preferences by considering environmental factors and achieve healthier body compositions. Therefore, the importance of such educational programs in promoting healthy lifestyles should be reemphasized. In this context, randomized controlled trials with larger and more diverse sample sizes and longer follow-up periods should be conducted, and emphasis should be placed on integrating sustainable nutrition education into routine dietary counseling.

### Strengths and limitations

This study has several strengths and weaknesses. First, it stands out as one of the limited randomized controlled trials examining the current definition of healthy and sustainable nutrition and the outcomes of nutritional interventions. Another strength was the one-on-one, face-to-face nature of the intervention, accompanied by a dietitian. The data obtained in this study were evaluated both over time and across groups, revealing all aspects of change. This was strengthened by the use of mixed-model repeated measures ANOVA, a method of analysis that was appropriate.

This study also has limitations. First, the study’s female participants may have led to gender bias. Furthermore, being conducted in a single center may limit the generalizability of the results. Furthermore, the self-reported measurement of dietary habits, sustainable eating behaviors, and some lifestyle parameters may have introduced bias into the data. Mediterranean diet adherence was encouraged through counseling; however, it was not quantitatively assessed using a validated adherence scale, which represents a limitation of the study.

## Data Availability

The datasets generated during and/or analyzed during the current study are available from the corresponding author on a reasonable request.

## Abbreviations

BIA: Bioelectrical impedance analysis
BMI: Body Mass Index
BSTSN: Behaviors scale towards sustainable nutrition
CONSORT: Consolidated Standards of Reporting Trials
IQR: Interquartile ranges
MD: Mean differences
SD: Standard deaviation
SDG: Sustainable Development Goals
